# Binding Mechanism of Inhibitors to CDK6 Deciphered by Multiple Independent Molecular Dynamics Simulations and Free Energy Predictions

**DOI:** 10.3390/molecules30050979

**Published:** 2025-02-20

**Authors:** Lifei Wang, Yan Wang, Lulu Zhang, Juan Zhao, Shiliang Wu, Zhiyong Yang

**Affiliations:** 1 School of Science, Shandong Jiaotong University, Jinan 250357, China; wanglf@sdjtu.edu.cn (L.W.); 211092@sdjtu.edu.cn (Y.W.); zhanglulu@sdjtu.edu.cn (L.Z.); zhaojuan@sdjtu.edu.cn (J.Z.); 211061@sdjtu.edu.cn (S.W.); 2 Department of Physics, Jiangxi Agricultural University, Nanchang 330045, China

**Keywords:** cyclin-dependent kinase 6, MM-GBSA, multiple independent molecular dynamics, GB models, principal component analysis

## Abstract

Cyclin-dependent kinase 6 (CDK6) has been identified as a potential drug target in various types of cancers. In our current study, multiple independent molecular dynamics simulations of four separate replicates and computations of binding free energies are carried out to decipher the binding mechanisms of three inhibitors, LQQ, 6ZV, and 0RS, to CDK6. The dynamic analyses indicate that the presence of inhibitors influences conformational alterations, motion modes, and the internal dynamics of CDK6. Binding free energies computed using the molecular mechanics generalized Born surface area (MM-GBSA) approach with four GB models demonstrate that hydrophobic interactions play essential roles in inhibitor–CDK6 binding. The computations of residue-based free energy decomposition verify that the side chains of residues I19, K29, M54, P55, F98, H100, and L152 significantly contribute to inhibitor–CDK6 binding, revealing the critical interaction sites of inhibitors for CDK6. The information revealed in our current study can provide theoretical aids for development of potent inhibitors targeting the CDK family.

## 1. Introduction

Cyclin-dependent kinases (CDKs) are a conserved eukaryotic group of serine/threonine kinases that play critical roles in progression of the cell or transcription cycles, metabolism, and apoptosis [1,2,3]. To date, research has recognized 20 CDKs in metazoans, which are intensely involved in the regulation of cellular processes. These kinases are divided into two subfamilies: the cell cycle CDKs (CDK1–6, 11, and CDK14–18) and the transcriptional cycle CDKs (CDK7–13, 19, and 20) [4,5,6]. As CDKs are related to monitoring genome stability and cell proliferation, they have been attractive therapeutic targets for the development of anticancer drugs [7,8]. Among the CDKs, cyclin-dependent kinase 6 (CDK6) is frequently found to be overexpressed in multiple types of human cancers and plays a pivotal role in regulating cell cycle progression [9,10]. Over the past few years, CDK6 has emerged as a prominent target for drug development, with the aim of the diagnosis and treatment of tumors. Moreover, numerous pharmacological inhibitors of CDK6 have been developed, showing promising activities in anticancer therapeutics by regulating cell survival, proliferation, cytokine production, and angiogenesis [11,12,13]. Therefore, insights into the binding mechanism of inhibitors to CDK6 is of paramount importance for design and development of potent inhibitors targeting CDK6.

CDK6 plays an essential role in initiating the cell cycle and exerts both positive and negative influences on cell cycle progression and tumor development [14,15,16,17]. Notably, the previous studies indicated that CDK6 expression was absolutely upregulated in patients with de novo Acute Myeloid Leukemia (AML) compared to healthy individuals [18]. Therefore, extensive works of research groups all over the world have been concentrating on devising novel, efficient small-molecule inhibitors targeting CDK6. For instance, abemaciclib, ribociclib, and palbociclib are three CDK6 inhibitors that have progressed to clinical diagnosis and treatment [19,20,21,22]. Additionally, three other inhibitors, namely, 7X, PD0183812, and AMG925, are in the preclinical phases and are undergoing disease screening and pharmaceutical development [23]. Most of these small-molecule inhibitors work by binding to the ATP-binding site of CDK6, inducing conformational alterations that prevent cell cycle progression as well as inhibit kinase-independent functions. The previous works indicated that the binding of inhibitors can induce alterations in the active and inactive states of the CDK family, which shows the roles of inhibitors in activation mechanism of the CDK family and drug design [24,25,26]. Thus, exploring conformational alterations of CDK6 due to the binding of these inhibitors and how they interact with the enzyme is significant for the development of novel, potent inhibitors targeting CDK6.

To effectively regulate and control the activity of CDK6, many investigators have accomplished experimental and computational studies to decode the binding mechanism of inhibitors to CDK6 and the other members of CDKs [27]. These works have provided valuable information about the inhibitor–CDK6 binding mechanism, which is crucial for drug development. For example, Yousuf et al. identified taurine as a novel CDK6 inhibitor using a fluorescence binding assay, isothermal titration calorimetry, and kinase assay, which confirmed the inhibition of the activity of CDK6 by taurine, and opened a new avenue to treat CDK6-associated cancers [28]. Additionally, research by Chen et al. on the role of miR-26a in multiple myeloma found that miR-26a could inhibit cell cycle progression by restraining the expression of CDK6, suggesting its potential for clinical applications [29]. Besides experimental studies, a variety of computational simulation works have been employed to probe the interactions between inhibitors and CDK6 [30]. For instance, He’s group applied structure-based and ligand-based computational approaches to clarify binding modes of CDK6 and its ligands, identifying two novel CDK6 inhibitors from the ChemBridge and ChemDiv compound libraries [31]. Dong et al. utilized an essential dynamic analysis and binding free energy calculations to assess the affinity of selonsertib for CDK6, suggesting its effectiveness as a CDK6 inhibitor [32]. In our previous work, we employed multiple short molecular dynamics (MD) simulations and depicted binding free energy surfaces to explore the binding selectivity of three inhibitors to CDK2 and CDK6 [33]. Sun et al. investigated binding selectivity of inhibitors to CDK4/6 over CDK1/2, and their results suggested that the molecular electrostatic potential (MESP) provides certain forces for the binding selectivity of inhibitors to different family members of CDKs [34]. In spite of these significant contributions, a deeper understanding of the molecular-level interactions between inhibitors and CDK6 is still essential for the design of more effective inhibitors targeting CDK6.

A range of calculation techniques [35,36,37,38,39], consisting of all-atom MD simulations [40,41], principal component analysis (PCA), dynamic cross-correlation maps [42], and free energy surfaces, have obtained great successes in illustrating the binding mechanisms of inhibitors to their targets. The integration of MD simulations with molecular docking has obtained great successes in providing insights into the binding mechanism of inhibitors to targets [43,44,45,46,47,48,49]. While conventional MD simulations followed by binding free energy calculations [50,51,52,53] have displayed significant potential in exploring conformational alterations in proteins induced by inhibitor binding, the advent of multiple independent MD simulations [54,55] has been proposed by several research groups. Compared to conventional MD simulations, multiple independent MD simulations more easily overcome the energy barrier and avoid the possibility of sampled conformations falling into local minimized spaces, which significantly enhances the sampling efficiency of target proteins.

**Figure 1:**
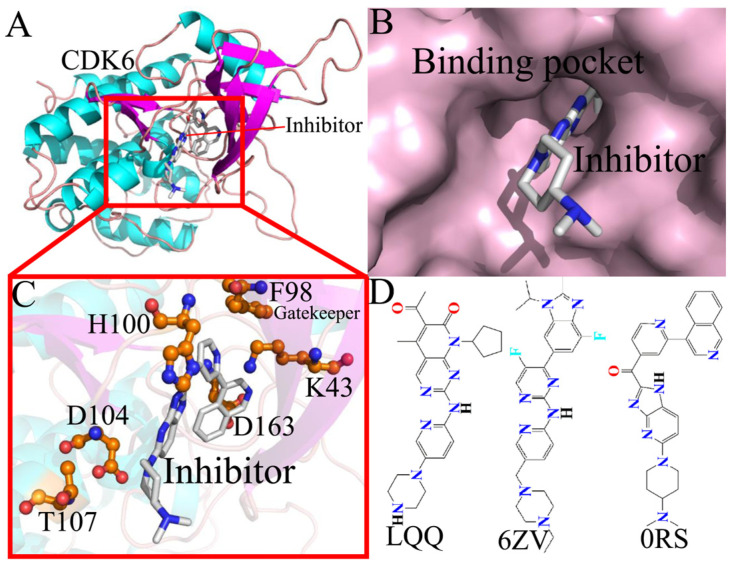
Molecular structures: (**A**) structure of the inhibitor–CDK6 complex, (**B**) binding pocket of CDK6 displayed in surface mode, (**C**) the ATP-binding site and gatekeeper of F98, and (**D**) structures of the three inhibitors, LQQ, 6ZV, and 0RS. In this figure, CDK6 is shown in cartoon mode, key residues are depicted in stick mode, and inhibitors are displayed in stick or line mode.

To unravel the effects of inhibitor associations on the conformational changes in CDK6, three small molecules, palbociclib (LQQ), abemaciclib (6ZV), and a 7-azabenzimidazole derivative (0RS), are selected to decipher their binding modes to CDK6. The binding ability of LQQ to CDK6 is scaled in an IC50 value of 16 nM, that of 6ZV to CDK6 in an IC50 value of 10 nM, while that of 0RS to CDK6 is in an IC50 value of 300 nM [31,56,57]. These data show their different binding abilities to CDK6, implying that it is essential to further study the molecular mechanisms of inhibitor–CDK6 binding for the development of small-molecule inhibitors for CDK6. The conformation and binding pocket of the inhibitor–CDK6 complex are displayed in Figure 1A–C, while the chemical structures of the three inhibitors are depicted in Figure 1D. Key hot spots on CDK6 are highlighted using stick representations, with a particular focus on the ATP-binding pocket, composed of residues L43, F98, H100, D104, T107, and D163. In the current study, multiple independent MD simulations and multiple analytical technologies, mainly including free energy calculations, PCA, free energy surface analysis, an examination of hydrophobic interactions (HIs), and analyses of intermolecular hydrogen bonds are integrated to decipher the binding mechanism between inhibitors and CDK6. Meanwhile, this work is expected to provide meaningful molecular binding dynamics and conformational shifts for the design of more effective CDK6 inhibitors.

## 2. Results and Discussion

### 2.1. Dynamic Equilibrium and Internal Dynamics of CDK6

**Figure 2:**
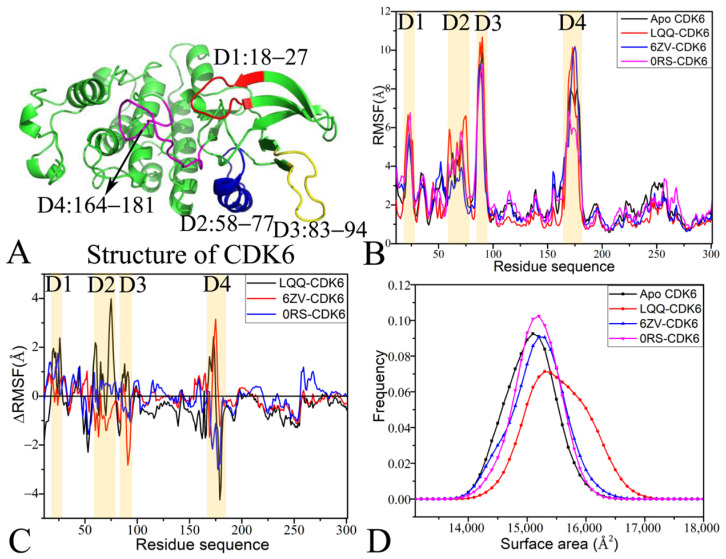
Structural flexibility and molecular surface areas of CDK6 in four systems: (**A**) structural domains (D1, D2, D3, and D4) of evident changes in RMSFs, (**B**) RMSFs of Apo, LQQ-, 6ZV- and 0RS-bound CDK6 used for scaling the structural flexibility of CDK6, (**C**) variations in RMSFs between Apo CDK6 and ligand-bound CDK6 used for evaluating changes in structural flexibility, and (**D**) molecular surface areas (MSAs) of Apo, LQQ-, 6ZV- and 0RS-bound CDK6 utilized for describing the extent of CDK6 contact with solvent.

Root-mean-square deviations (RMSDs) and root-mean-square fluctuations (RMSFs) are key indicators for evaluating structural stability and flexibility. To estimate the whole stability of CDK6 during four individual MD simulations, RMSDs of the backbone atoms in CDK6 compared with the initially optimized conformations were computed as time evolved and are displayed in Figure S1. The structural fluctuations of four replicas of all systems reached equilibrium after 100 ns of MD simulations. Thus, the stable parts (100–600 ns) from four independent MD simulations were linked together to constitute a single integrated trajectory (SIT) of 2.0 μs for every complex, which was applied to implement subsequent calculations and dynamic analyses. The structural domains related to obvious alterations in the RMSFs are displayed in Figure 2A. Evident structural changes primarily exist in four regions, consisting of D1 (residues 18–27), D2 (residues 58–77), D3 (residues 83–94), and D4 (residues 164–181). The RMSFs of the C_α_ atoms from CDK6 were calculated on the basis of the SIT to assess the structural flexibility of CDK6 (Figure 2B). It was found that the RMSFs of the four domains, D1, D2, D3, and D4, were markedly higher than those in other regions, indicating significant flexibility in these domains. Furthermore, we calculated the RMSF differences between the Apo and ligand-bound CDK6 complexes using the formula $∆RMSF={RMSF}_{bound}-{RMSF}_{Apo}$, in which ∆RMSF denotes the change in RMSFs, and ${RMSF}_{bound}$ and ${RMSF}_{Apo}$ represent the RMSFs of the bound and Apo forms of CDK6, respectively (Figure 2B). The association of LQQ and 0RS significantly increased the flexibility of domain D1, while 6ZV had a slight effect on this domain’s flexibility (Figure 2C). Similarly, LQQ and 0RS binding enhanced the RMSFs of domain D2, whereas 6ZV binding led to a noticeable reduction in the flexibility of this domain. This suggests that LQQ and 0RS binding promotes flexibility in D2, while the 6ZV association diminishes it. Moreover, LQQ binding was found to increase the flexibility of domains D3 and D4 relative to the Apo form, whereas 0RS binding reduced the flexibility of these domains. Contrarily, 6ZV binding significantly reduced the flexibility of domain D3 but enhanced that of D4. Based on the current analysis, the domains involved in obvious changes in the structural flexibility of CDK6 imply key residues participating in inhibitor–residue interactions, which is in basic agreement with the calculations of residue-based free energy decomposition.

Molecular surface areas (MSAs) are frequently employed to represent the extent of whole solvent-accessible contacts of receptors. In our present work, the MSAs of Apo, LQQ-, 6ZV- and 0RS-bound CDK6 were computed with the SIT and the corresponding frequency distributions are shown in Figure 2D. The computed MSAs reveal an increase in surface areas for the LQQ-, 6ZV-, and 0RS-bound CDK6 complexes by 200, 100, and 100 Å^2^, individually, in comparison to the Apo form of CDK6. According to Figure 2D, the MSAs of the LQQ-bound CDK6 are greater than those of all other simulated systems, mainly due to the higher flexibility of the D1, D2, and D3 loops in the protein (Figure 2B,C). This enlarges the extent of CDK6 contact with the solvent upon inhibitor binding, significantly impacting its interactions with the solvent environment and regulating the activity of CDK6.

**Figure 3:**
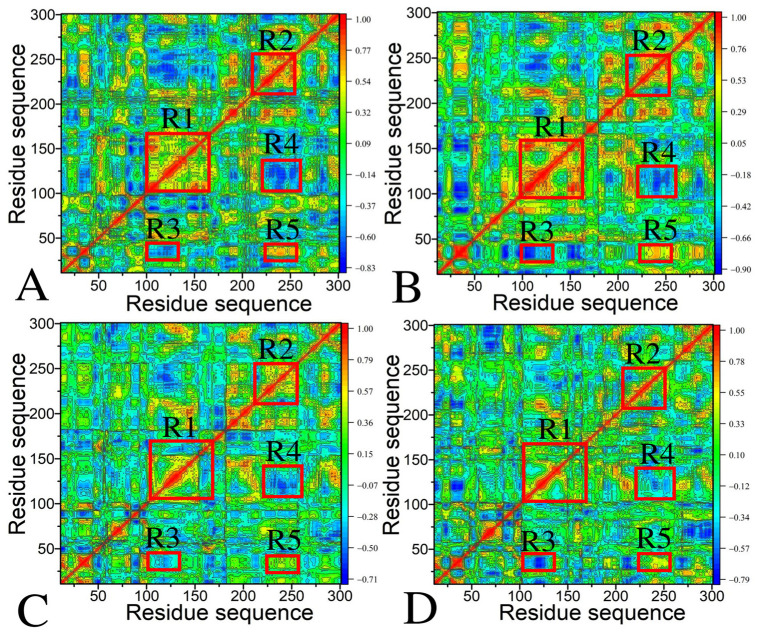
Dynamic cross-correlation maps computed by utilizing the coordinates of $C_{\alpha }$ atoms around their mean positions recorded at the single joined trajectory: (**A**) Apo CDK6, (**B**) LQQ-bound CDK6, (**C**) 6ZV-bound CDK6, and (**D**) 0RS-bound CDK6.

To assess the influence of ligand binding on the internal dynamics of CDK6, dynamic cross-correlation maps (DCCMs) were generated using the coordinates of the C_α_ atoms from the single integrated trajectory. The outcomes are visually represented in Figure 3, where color-coded interpretations provide insights into the motion correlations among residues. Specifically, areas shaded in yellow and red illustrate regions of extremely positive correlated motions (PCMs), indicating synchronous movement among certain residues. Conversely, areas in blue and dark blue highlight strongly anticorrelated movements (ACMs), reflecting opposite motion directions between residue pairs.

For Apo CDK6 (Figure 3A), a few intensely correlated movements are displayed: (1) two diagonal regions, R1 and R2, generate the obvious PCMs of residues 100–168 and 202–260 from CDK6 relative to themselves, (2) region R3 characterizes the ACMs of residues 102–137 relative to the N-terminus of CDK6, (3) region R4 also demonstrates strong ACMs between residues 222–263 and 101–145, and (4) region R5 embodies the PCMs of residues 221–261 relative to the N-terminus of CDK6.

The internal dynamics and movement patterns of CDK6 undergo significant changes upon binding to the three inhibitors, as depicted in Figure 3B–D. According to Figure 3B, the binding of LQQ not only strengthens the PCM of region R1 and the ACM of region R3 but also highly weakens the PCMs of regions R2 and R5 and the ACM of region R4. Similarly, the binding of the other two inhibitors, 6ZV and 0RS, exerts comparable effects on the dynamic behavior of CDK6. Both associations lead to a reduction in the PCMs of regions R1, R2, and R5 and a weakening of the ACMs in region R4, as illustrated in Figure 3C, D. These observations reveal that each inhibitor, despite their varying chemical structures, induces distinct alterations in the internal dynamics of CDK6. The alterations highlight the nuanced impact of inhibitor binding on the movement patterns within the protein, indicating the complexity of the interaction mechanisms and the potential for tailored therapeutic interventions based on these dynamic changes. The aforementioned structural regions not only agree with the previous RMSF analysis but also reveal hot interaction spots of inhibitors with CDK6.

### 2.2. Alterations in the Dynamic Behaviors of CDK6 Due to Inhibitor Associations

In the drug development process, understanding the conformational and dynamic alterations of target proteins is paramount. PCA is applied to the SITs of four simulated systems to investigate the conformational changes in CDK6, involving the diagonalization of a covariance matrix constructed using the C_α_ atomic coordinates retained from the multiple independent MD trajectories. In the present study, PCA was adopted to obtain eigenvalues and eigenvectors by means of the CPPTRAJ program in Amber 20. The eigenvalues extracted from the PCA were applied to embody the conformational fluctuation of CDK6 along the eigenvectors. The function of eigenvalues relative to their corresponding eigenvector indices, depicted in Figure S2, reveals significant insights into the conformational variability of CDK6. Notably, the first six eigenvalues cumulatively capture a substantial portion of the overall motion for each state of CDK6: 90.73% for the Apo form, 93.46% for LQQ-bound CDK6, 86.12% for 6ZV-bound CDK6, and 88.97% for 0RS-bound CDK6. This quantification underscores the extent to which these initial eigenvalues account for the majority of the protein’s dynamic behavior, indicating that the binding of each inhibitor distinctly influences CDK6’s conformational landscape.

**Figure 4:**
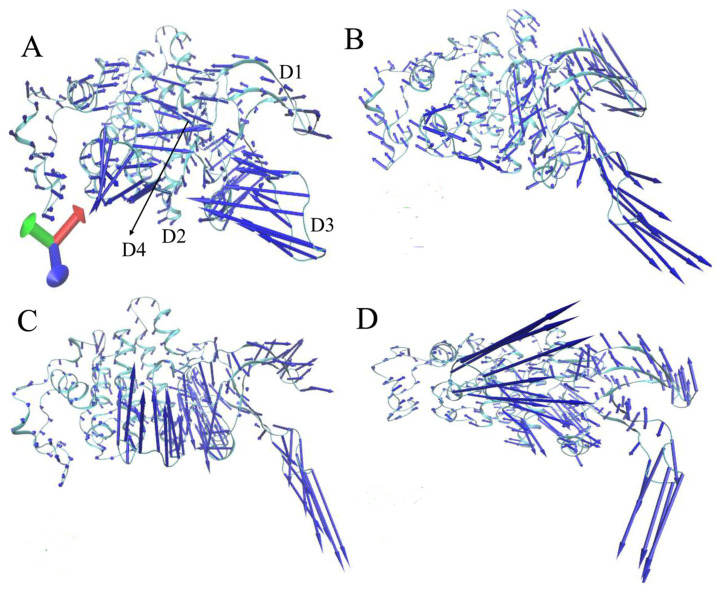
Collective motions of the domains in CDK6 displayed using the first eigenvector from PCA: (**A**) Apo CDK6, (**B**) LQQ-bound CDK6, (**C**) 6ZV-bound CDK6, and (**D**) 0RS-bound CDK6. The direction and length of the arrows characterize the direction and strength of the domain motions.

The orientation and amplitude of movements within CDK6’s secondary structure, influenced by the binding of inhibitors LQQ, 6ZV, and 0RS, were analyzed using eigenvectors obtained from the PCA. These dynamics are visually represented in porcupine plots (Figure 4), providing insights into the concerted motions of CDK6’s domains upon the inhibitor interaction. It was found that the presence of LQQ, 6ZV, and 0RS influences the concerted motion of CDK6. Compared to Apo CDK6 (Figure 4A), the association of LQQ, 6ZV, and 0RS both altered the inclination of the concerted motion within the D1 domain (residues 18–27) and abated the fluctuation amplitude of this domain (Figure 4B–D). The association of 0RS changed the orientation of the concerted movements in the D2 domain (residues 58–77). For the D3 domain (residues 83–94), the presence of LQQ, 6ZV, and 0RS not only shifted the direction of its concerted motions but also increased the fluctuation amplitude, indicating enhanced dynamic flexibility compared to the Apo form (Figure 4B–D). The gatekeeper residue F98 is situated in domain D3; thus, the changes in flexibility of D3 certainly indicate the dynamic behavior of the gatekeeper. Similarly, the D4 domain (residues 164–181) experiences changes in the motion inclination and an amplification of fluctuation amplitude upon the association of 6ZV and 0RS, as opposed to the Apo CDK6 (Figure 4C,D). These observations reveal that the binding of each inhibitor distinctly affects the motion orientations and amplitudes within specific domains of CDK6, underscoring the nuanced impacts of inhibitor associations on the enzyme’s internal dynamics. Such insights are crucial for understanding the mechanistic basis of inhibitor-induced alterations and their implications for drug development targeting CDK6.

**Figure 5:**
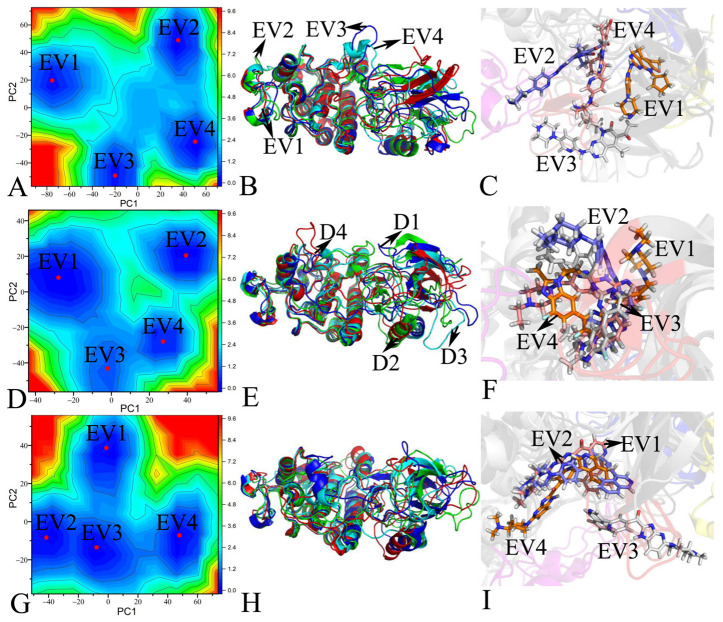
Free energy surfaces and structural information: (**A**) free energy surfaces of LQQ-bound CDK6, (**B**) structural superimpositions of LQQ-bound CDK6 situated at EV1-EV4, (**C**) structural alignment of LQQ located at EV1-EV4, (**D**) free energy surfaces of 6ZV-bound CDK6, (**E**) structural superimpositions of 6ZV-bound CDK6 located at EV1-EV4, (**F**) structural alignment of 6ZV falling into the EV1-EV4 range, (**G**) free energy surfaces of 0RS-bound CDK6, (**H**) structural superimpositions of 0RS-bound CDK6, and (**I**) structural alignment of 0RS trapped at EV1-EV4. CDK6 and the three inhibitors, LQQ, 6ZV, and 0RS, are displayed in cartoon and stick modes, respectively.

To investigate the energetic aspects of the changes in CDK6’s concerted movements resulting from inhibitor binding, free energy surfaces (FESs) were constructed by means of the projections of the SIT onto the first two principal components (PC1 and PC2) as reaction coordinates (RCs). The FESs, along with pertinent structural insights, are depicted in Figure 5 and Figure S3. The FESs reveal that the binding of the inhibitors LQQ, 6ZV, and 0RS notably modifies CDK6’s free energy profiles, facilitating conformational rearrangements within the protein. As illustrated in these figures, the association of each inhibitor leads to distinct alterations in the energy landscape, highlighting the diverse pathways through which these compounds influence CDK6’s structural dynamics.

With regard to Apo CDK6, four independent MD simulations detected four distinct energy valleys (EV), marked as EV1, EV2, EV3, and EV4 (Figure S3). This result indicates that the conformations of Apo CDK6 are primarily distributed in four conformational subspaces. This observation suggests that Apo CDK6’s conformations predominantly occupy four separate conformational subspaces, indicating a degree of structural diversity within its unbound state. Superimposition of four representative structures from these energy wells, as shown in Figure S3B, reveals significant deviations across the structural domains D1, D2, D3, and D4, with particularly notable variations in the D1 and D4 domains. Upon inhibitor binding, there is a noticeable reduction in deviations within the D1 domain, as evidenced in Figure 5B,E,H, suggesting that the inhibitors stabilize this region of CDK6. Moreover, the interactions with LQQ and 6ZV lead to a convergence in the structure of the D2 domain, indicating a unification of conformations that may be critical for the protein’s activity (Figure 5B,E). Similarly, the binding of LQQ and 0RS results in diminished deviations in the D4 domain (Figure 5B,H), further pointing to the selective impacts of these inhibitors on CDK6’s structural dynamics. The alterations induced by the binding of the inhibitors LQQ, 6ZV, and 0RS not only involve changes in structural deviations but also introduce notable sliding and twisting motions (Figure 5C,F,I). These movements significantly contribute to the dynamic association between CDK6 and its inhibitors, highlighting the complex interplay between the structural conformation and inhibitor efficacy. Based on the structural superimposition of three inhibitors located at different EVs, four distinct binding poses are observed. It is found that the binding poses of the three inhibitors vary significantly within the binding pocket of CDK6, altering the orientations of the inhibitors relative to the binding regions of CDK6 and influencing their binding to CDK6.

Based on the above discussions, the interaction of the inhibitors with CDK6 significantly alters the protein’s internal dynamics, particularly affecting the correlated movements between its conformational domains. These changes influence not only the concerted movements within the structural domains D1–D4 of CDK6 but also the dynamic behaviors of D1–D4; moreover, D1–D4 are involved in the binding of inhibitors and the catalytic activity of CDK6. Therefore, they directly impact the binding efficacy and specificity of the inhibitors towards CDK6. The relative positions of the three inhibitors in CDK6 are displayed in Figures S4–S6 in separate modes, showing different orientations within the binding pocket. According to Figure 5 and Figures S4–S6, although 6ZV exhibits four distinct binding poses in CDK6, its structural deviation is smaller than that of LQQ and 0RS, implying that 6ZV possesses higher structural stability compared to the other two inhibitors. It is also observed that the structural deviation of LQQ in the binding pocket is smaller than that of 0RS. These results suggest that 6ZV should exhibit the strongest binding to CDK6. Furthermore, our current findings align with the subsequent results of the binding free energy calculation. This interplay between CDK6’s conformational dynamics and inhibitor binding highlights the importance of understanding protein flexibility and movement modes in the drug development of targeted therapeutics.

### 2.3. Binding Free Energy Computations by MM-GBSA and a Molecular Docking Approach

To estimate the binding ability of the inhibitors LQQ, 6ZV, and 0RS to CDK6, the MM-GBSA approach was adopted to compute the binding free energies of the three inhibitors to CDK6 using 500 snapshots stemming from the 2.0 μs SIT with a time step of 4 ns. Given the computational intensity associated with entropy calculations, a subset of 100 snapshots was extracted from above-mentioned 500 snapshots to specifically assess the entropy contributions to the binding energies between the inhibitors and CDK6. The MM-GBSA approaches are possibly related to various generalized Born (GB) models. Yang and Wang et al. adopted different GB models to calculate the binding free energies of inhibitors to targets, and their results indicated that the selection of the GB model generates a large effect on the prediction of binding free energies [58,59]. Thus, to investigate the influence of the different GB models on the binding affinities, four different GB models, recognized by IGB = 1, IGB = 2, IGB = 5, and IGB = 66, were selected to compute the binding free energies of three inhibitors to CDK6. Key parameters for the MM-GBSA calculations with these models, including two empirical parameters (γ and β) and the radii types, are summarized in Table S1. The resulting binding free energies, along with their separate components, are displayed in Table 1. These data provide detailed insights into the binding dynamics and energetic profiles of the inhibitor–CDK6 associations, underlining the influence of different GB models on the estimated binding affinities.

Binding free energies primarily consist of five components, including electrostatic interactions ($∆E_{ele}$), van der Waals interactions ($∆E_{vdw}$), polar solvation free energy ($∆G_{gb}$), non-polar solvation free energy ($∆G_{surf}$), and entropy contributions (−T∆S), which are listed in Table 1. Among the free energy components, $∆E_{ele}$, $∆E_{vdW}$, and $∆G_{surf}$ typically facilitate the binding of inhibitors to CDK6, contributing positively to the association energy. Conversely, the other two sections, $∆G_{gb}$ and (−T∆S), prevent inhibitor–CDK6 binding. Notably, the combination of $∆E_{vdW}$ and $∆G_{surf}$ underpins the hydrophobic interactions between the inhibitors and CDK6, playing a critical role in stabilizing the inhibitor–protein complex. Meanwhile, the polar interactions $∆G_{pol}$, arising from the sum of $∆E_{ele}$ and $∆G_{gb},$ yield a distinct force on the inhibitor–CDK6 association. An analysis based on different GB models (IGB = 1, IGB = 2, and IGB = 66) suggests that these polar interactions typically oppose the binding of 6ZV and 0RS to CDK6. In contrast, the results from the GB model IGB = 5 indicate a favorable contribution to the binding of these inhibitors. The rank of binding free energies determined by molecular docking also aligns with that determined by MM-GBSA calculations and the experimental values, further indicating the reliability of our results.

To evaluate the results of MM-GBSA calculations, LQQ, 6ZV, and 0RS were docked into the binding pocket of CDK6 to determine their binding affinities (Table S2). The docked structures corresponding to the first twenty docking values are illustrated in Figure S4. The binding strengths of the three inhibitors, ranked by the first six values, are in the order 6ZV > LQQ > 0RS, which is consistent with the both the MM-GBSA calculations and the experimental values. These results indicate that our MM-GBSA calculations based on multiple independent MD simulations are dependable. Figure S7 demonstrates that LQQ, 6ZV, and 0RS possess five, four, and five binding sites, respectively.

Moreover, the binding enthalpy (∆H), incorporating $∆E_{ele}$, $∆E_{vdw}$, $∆G_{gb}$, and $∆G_{surf}$, exerts a beneficial influence on the inhibitor–CDK6 associations. The choice of GB models in MM-GBSA calculations prominently obviously affects the polar solvation free energy ($∆G_{gb}$). Additionally, the selection of the empirical parameters γ and β significantly influences the determination of non-polar solvation free energies ($∆G_{surf}$), as displayed in Table 1.

Among the four different GB models, the binding free energies of LQQ, 6ZV, and 0RS to CDK6 computed with the GB model of IGB = 2 are closest to the experimental observations. Therefore, the parameters of IGB = 2 yield more rational results, as verified by previous works [60,61]. While the ranking of predicted binding free energies with the models of IGB = 1 and IGB = 66 align with the experimental data, the numerical values significantly diverge. Conversely, the ranking derived from the model of IGB = 5 is not consistent with the experimental findings, indicating its lesser predictive accuracy for this set of inhibitors. Based on comparison with the experimental values, the results from the IGB = 2 model are more reliable than those of the three other models [31,56,57]. Therefore, the results computed by means of the GB model IGB = 2 were applied to evaluate the binding ability of LQQ, 6ZV, and 0RS to CDK6. The electrostatic interactions of LQQ and 6ZV with CDK6 were decreased by 12.97 and 13.13 kcal/mol in comparison to those of 0RS with CDK6, independently. Meanwhile, the unfavorable polar solvation free energies of the LQQ- and 6ZV-CDK6 complexes were reduced by 8.99 and 8.77 kcal/mol compared to those of the 0RS-CDK6 complex. On the whole, the polar interactions of LQQ and 6ZV with CDK6 were improved by 3.98 and 4.36 kcal/mol in contrast with that of the 0RS-CDK6 complex. The hydrophobic interactions of LQQ and 6ZV with CDK6 were increased by 6.27 and 10.33 kcal/mol, respectively, versus the 0RS-CDK6 complex. On the whole, the enthalpy contributions to the LQQ- and 6ZV-CDK6 complexes were enhanced by 2.29 and 5.97 kcal/mol compared to those of the 0RS-CDK6 complex. Moreover, the unfavorable entropy contributions to LQQ- and 6ZV-CDK6 binding were strengthened by 1.16 and 0.83 kcal/mol compared with the 0RS-CDK6 complex. In summary, the binding affinity of LQQ and 6ZB to CDK6 was enhanced by 1.13 and 5.14 kcal/mol in contrast to that of 0RS to CDK6 (Table 2). Overall, these computational insights reveal that despite the minor structural differences among the inhibitors, their binding affinities for CDK6 exhibit notable variations, which could be induced by conformational changes relative to inhibitor associations.

Based on the above analyses, our results verify that the model of IGB = 2 can obtain more rational results than the three other models in predicting the binding free energies of inhibitors to CDK6, which provides a scheme for studying the binding of inhibitors to CDK6. Meanwhile, van der Waals interactions play significant roles in inhibitor–CDK6 binding, which is a main factor to be considered in future drug design targeting CDK6.

### 2.4. Analyses of Ligand–CDK6 Interaction Networks

**Figure 6:**
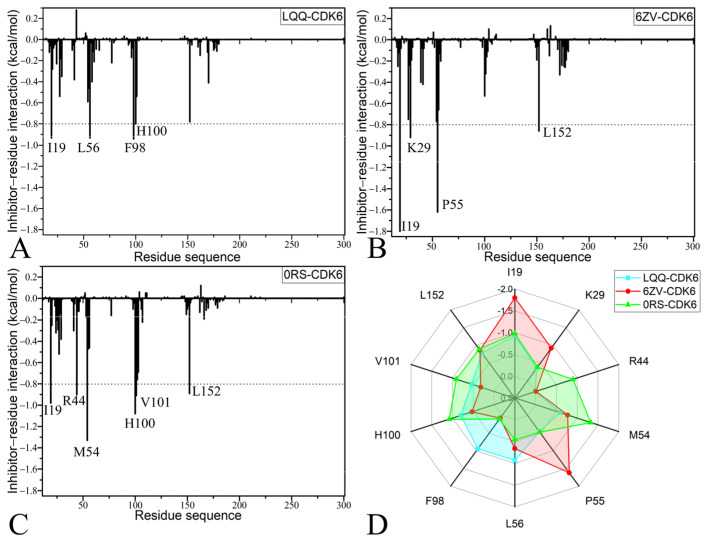
Interactions of the inhibitors with CDK6: (**A**) interaction spectrum of LQQ with each residue of CDK6, (**B**) interaction spectrum of 6ZV with each residue of CDK6, (**C**) interaction spectrum of 0RS with each residue of CDK6, and (**D**) important residues involved in the binding of LQQ, 6ZV, and 0RS to CDK6.

**Figure 7:**
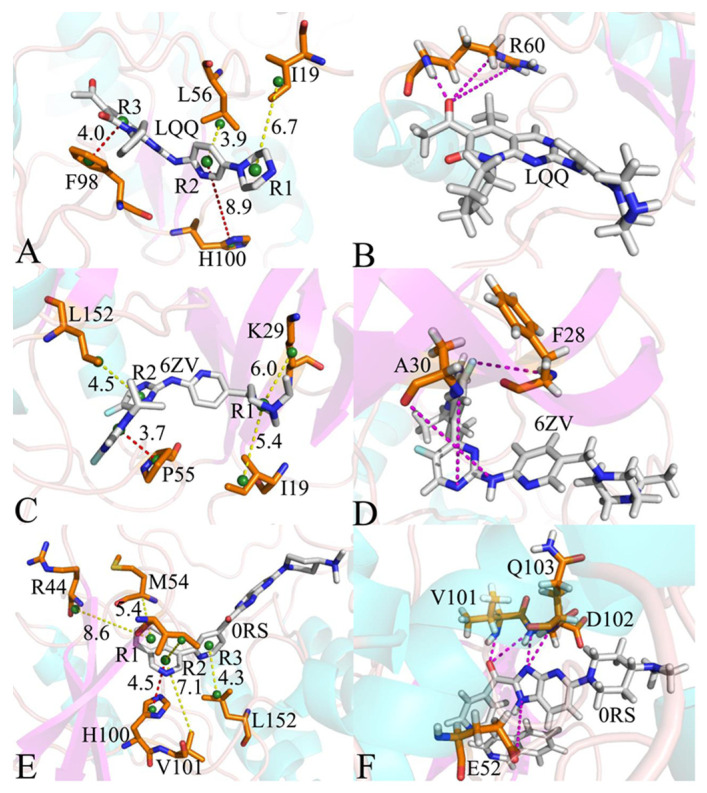
Geometric structures of inhibitor–residue interactions: (**A**) the hydrophobic interactions between LQQ and important residues, (**B**) the LQQ-CDK6 HBIs, (**C**) the hydrophobic interactions of 6ZV with important residues, (**D**) the 6ZV-CDK6 HBIs, (**E**) the hydrophobic interactions of 0RS with important residues, and (**F**) the 0RS-CDK6 HBIs. The yellow dashed lines describe the CH-π interactions, the red dashed lines indicate the π-π interactions, and the magenta dashed lines represent the HBIs.

To probe the roles of separate amino acids in the binding affinity of inhibitors, the residue-based free energy decomposition approach was applied to assess the interactions between three inhibitors (LQQ, 6ZV, and 0RS) and the respective residues of CDK6 (Figure 6A–C). The energetic contributions from the side chains and backbones of pivotal residues to the binding free energies between inhibitors and CDK6 are given in Table 2. Additionally, hydrogen bonding interactions (HBIs) of ligands with CDK6 were analyzed using the CPPTRAJ module within Amber 20, and the results are provided in Table 3. Furthermore, the configuration information of hydrophobic interactions and HBIs between the inhibitors and key residues are illustrated in Figure 7, providing a geometric representation of these critical molecular interactions.

In the case of the LQQ-CDK6 complex, four specific residues of CDK6, namely, I19, L56, F98, and H100, have energy contributions exceeding −0.8 kcal/mol to LQQ-CDK6 binding (Figure 6A,D). As shown in Figure 7A, the alkyl groups or carbon atoms of residues I19 and L56 are adjacent to the hydrophobic rings R1 and R2 of LQQ, which yield the CH-π interactions between them. According to Table 2, the binding energies of I19 and L56 with LQQ are both −0.93 kcal/mol, which mainly provided by the side chains of these residues. Meanwhile, the phenyl group of F98 and the ring of H100 generate the π-π interactions with the hydrophobic rings R2 and R3 of LQQ, which are marked by red dashed lines in Figure 7A. The energy contributions of F98 and H100 to LQQ-CDK6 binding are −0.94 and −0.80 kcal/mol, respectively, which are primarily derived from van der Waals interactions between side chains, while the latter results come from a composite of van der Waals and electrostatic interactions between the side chains and backbone, respectively. The interaction energies of F98 and H100 with LQQ are −0.94 and 0.80 kcal/mol, respectively; moreover, the F98-LQQ binding energy mainly stems from the van der Waals interactions of the side chains, while the H100-LQQ interaction energy comes from the sum of van der Waals interactions of the side chains and electrostatic interactions of the backbone (Table 2). Additionally, the amino group of R60 establishes three HBIs with the O01 atom of LQQ, and their occupancies are 33.57%, 26.31%, and 17.97%, separately (Table 3 and Figure 7B).

Regarding the 6ZV-CDK6 complex, four residues, specifically, I19, K29, P55, and L152, provide energy contributions exceeding −0.8 kcal/mol toward the binding affinity with 6ZV, as depicted in Figure 6B,D. The structural analysis reveals that the alkyl groups or carbon atoms of residues I19 and K29 are adjacent to the hydrophobic ring R1 of 6ZV, facilitating the CH-π interactions. These interactions are illustrated in Figure 7B and are quantified to contribute interaction energies of −1.80 kcal/mol and −0.92 kcal/mol, respectively. Similarly, the ring of P55 and the alkyl groups of L152 are involved in the CH-π interactions, with corresponding interaction energies of −1.62 kcal/mol and −0.86 kcal/mol, as detailed in Figure 7C and Table 3. Notably, the interaction energies of 6ZV with I19, P55, and L152 predominantly arise from van der Waals forces attributable to the side chain interactions, whereas the engagement of K29 with 6ZV is derived from a combination of van der Waals and electrostatic interactions pertaining to the side chains. Moreover, according to Table 3, residues A30 and F28 form three HBIs with 6ZV, exhibiting occupancies of 21.51%, 20.64%, and 12.23%, individually.

With respect to the 0RS-CDK6 compound, the interactions of 0RS with six residues in CDK6 are higher than −0.8 kcal/mol, and these residues comprise I19, R44, M54, H100, V101, and L152 (Figure 6C,D). The interaction energies of I19, R44, M54, V101, and L152 with 0RS are −0.98, −0.90, −1.33, −0.91, and −0.89 kcal/mol, independently, which structurally agree with the CH-π interactions of alkyl or carbon atoms of these five residues with rings R1, R2, and R3 of 0RS (Figure 7E). The analysis reveals that the energetic contributions from I19, M54, and L152 toward the 0RS-CDK6 complex predominantly emanate from van der Waals interactions with the side chains of the three residues. The interaction energies of R44 with 0RS mainly originate from the electrostatic interactions of its side chains, while the energy contribution of V101 to the 0RS-CDK6 association is chiefly provided by the electrostatic interactions of the backbone. Moreover, the carbonyl group of residue V101 not only generates hydrophobic interactions with ring R2 of 0RS but also forms an HBI with an occupancy of 27.35%. Additionally, residue H100 produces an interaction of −1.08 kcal/mol with 0RS (Table 3), which is in good agreement with the π-π interaction of the hydrophobic ring of H100 with ring R2 of 0RS (Figure 7E). Furthermore, the energy contributions mostly come from the van der Waals interactions of the side chains and electrostatic interactions of the backbone from H100 with the ring R2 of 0RS. According to Table 3, three residues, E52, D102, and Q103, form four HBIs with 0RS, and the corresponding occupancies are 24.81%, 18.40%, 17.98%, and 14.63%, respectively. The key residues revealed in this work are in basic agreement with previous studies [56,57]. Meanwhile, energy contributions provide key residues and support the previous binding free energy calculations. In addition, the key residues I19, K29, M54, P55, F98, H100, and L152 identified in this study can be used as efficient targets of drug design targeting CDK6. Based on our analyses of HBIs, the occupancy of hydrogen bonds, except for R60-NE-HE···LQQ-O01, is lower than 30%, which reflects the effect of the difference in the structures of inhibitors on the stability of hydrogen bonds.

## 3. Materials and Methods

### 3.1. Initialization of Simulated Systems

The initial structures of CDK6 bound to the inhibitors LQQ, 6ZV, and 0RS were sourced from the Protein Data Bank (PDB), with PDB entries 5L2I, 5L2S, and 4EZ5, respectively [31,56,57]. To obtain the Apo form of CDK6 (the enzyme without any bound inhibitor), the LQQ inhibitor was removed from the 5L2I crystal structure. Since several residues are missing in the crystal structure of CDK6, the program MODELLER was adopted to renovate the lost residues in the crystal structures 5L2I, 5L2S, and 4EZ5 [62]. Due to sequence variations among the three compounds, residues 11–301 of CDK6 were applied to model the initial structures. The PROPKA method was applied to determine the protonation states of CDK6 residues, ensuring that each residue was correctly protonated [63,64]. Missing hydrogen atoms were added to the structures using the Leap module of Amber 20 [65,66]. The simulation parameters for CDK6 were assigned using the *ff*19SB force field [67]. The chemical structures of the inhibitors LQQ, 6ZV, and 0RS were optimized by means of the semi-empirical AM1 method, and, subsequently, the atomic bond charge correction (BCC) charges of LQQ, 6ZV, and 0RS were assigned using the Antechamber module of Amber 20. The general amber force field (GAFF) was implemented to produce the force field parameters of the three inhibitors, LQQ, 6ZV, and 0RS [68]. To simulate a physiological salt environment, 45 chloride ions (Cl^−^) and 46 sodium ions (Na^+^) were added to create a neutral system with a 0.15 M NaCl concentration for each compound, in which the parameters of Na+ and Cl- were obtained from the work of Li et al. [69]. The Apo CDK6 structure and the CDK6–inhibitor complexes were immersed in octahedral periodic boxes filled with the TIP3P water molecules of ~13,500, extending 10.0 Å in each direction, to facilitate the simulations.

### 3.2. Multiple Independent MD Simulations

To ensure the effectiveness of multiple independent MD simulations, all initial systems were subjected to two-step energy minimizations to eliminate high-energy contacts and repulsive orientations between atoms, including a 4000 step steepest descent minimization process and another 6000 step conjugate gradient one. Then, a slow heating process was carried out from 0 to 300 k in 3 ns in a constant number, volume, and temperature (NVT), in which all heavy atoms in CDK6 and the small-molecule inhibitors were restrained in a weak harmonic restriction of 2 kcalmol^−1^·Å^2^. Subsequently, a 3 ns equilibrium phase at 300 K was implemented on the four CDK6-related systems using the isothermal–isobaric ensemble (NPT) to further optimize the systems. Finally, four independent 600 ns MD simulations were performed on Apo CDK6, LQQ-, 6ZV-, and 0RS-bound CDK6 to deeply relax each system, and the simulation time of each system achieved 2.4 μs, through which the conformations were recorded with time intervals of 2 ps. Throughout these simulation stages, the Langevin equation, with a moderate damping coefficient of 2.0 ps^−1^, was utilized to regulate the temperature of the simulation systems [70]. The SHAKE algorithm was utilized to constrain the lengths of bonds involving hydrogen and heavy atoms in CDK6 at every trajectory step [71]. And the long-range electrostatic interactions were addressed using the smooth particle mesh Ewald approach with a 10 Å cutoff distance [72]; this cutoff was also used for calculating van der Waals interactions. For a comprehensive analysis, the equilibrium segments from the four independent conventional MD trajectories were merged into a single integrated trajectory. In the present work, all simulations were performed utilizing the pmemd.cuda program within the Amber 20 software package, leveraging its computational efficiency and accuracy [73,74].

### 3.3. MM-GBSA Free Energy Calculations and Decomposition

In this study, the molecular mechanics generalized Born surface area (MM-GBSA) and molecular mechanics Poisson–Boltzmann surface area (MM-PBSA) methods are highlighted as robust techniques for evaluating ligand binding affinities. Compared to the thermodynamic integration (TI) and free energy perturbation (FEP) methods, the accuracy of MM-PBSA and MM-GBSA are weaker, but TI and FEP are highly expensive. Specifically, Hou’s group has validated the accuracy and effectiveness of these approaches in predicting binding affinities of various small molecules to proteins, with MM-GBSA showing particularly promising results [75,76,77]. Consequently, we employed the MM-GBSA approach to calculate the binding free energies of the inhibitors LQQ, 6ZV, and 0RS to CDK6, as detailed in Equation (1):

(1) $$\Delta G_{bind}=\Delta E_{ele}+\Delta E_{vdw}+\Delta G_{gb}+\Delta G_{nonpol}-T\Delta S$$

where the first two terms $\Delta E_{ele}$ and $\Delta E_{vdw}$ represent the electrostatic and van der Waals interactions between the inhibitors and CDK6, which were calculated using the *ff*19SB force field. The components $\Delta G_{gb}$ and $\Delta G_{nonpol}$ correspond to the polar and nonpolar components of solvation free energy, separately. The polar component was evaluated using the GB model developed by Onufriev et al. [78], while the nonpolar component was derived from empirical Equation (2):

(2) $$\Delta G_{nonpol}=\gamma \times \Delta SASA+\beta$$

in which the parameters γ and β are individually set as 0.0072 kcal/(mol·Å^−2^) and 0.0 kcal/mol in the current work [79]. The term −TΔS accounts for the entropy contributions to the binding free energies, which were calculated using the mmpbsa_py_nabnmode in Amber 20.

### 3.4. Molecular Docking

Molecular docking, as one of the commonly employed virtual screening techniques, seeks to predict the binding conformations of small-molecule ligands at their specific target binding sites. The crystal structure of Apo CDK6 obtained from 5L2I served as the initial structure for docking the compounds LQQ, 6ZV, and 0RS into CDK6. And the docked structures were optimized to alleviate the high-energy interactions among the atoms. In our study, the grid box dimensions in the (x, y, z) directions were set to (60, 60, 60) Å, with a spacing of 0.375 Å. We utilized the Lamarckian genetic algorithm (LGA) along with the default parameters of the software to implement our present docking study. The first twenty binding free energies are displayed in Table S2 for comparison with the previous MM-GBSA results. The docked structures of the inhibitor–CDK6 complexes with the first twenty maximum values were presented in Figure S4, illustrating various binding poses. Molecular docking was conducted using AutoDock 4.2 [80,81].

### 3.5. Principal Component Analysis Methodology

PCA is an important tool for identifying large coordinated movements from a collection of conformational structures arising from molecular simulations or experiments. This methodology has been extensively used to investigate the conformational changes of receptors in relation to their function. PCA can be computed by diagonalizing a covariance matrix created from the atomic coordinates obtained in multiple independent MD simulations, resulting in a set of eigenvectors and eigenvalues. The eigenvectors indicate the motion directions within the conformational space of protein domains, while the eigenvalues signify the mean square fluctuations of movement along the respective eigenvectors. The initial eigenvectors with significant eigenvalues are particularly notable for illustrating the overall movements of proteins. In this study, the CPPTARJ module integrated within Amber was utilized to perform PCA on the combined multiple independent MD trajectories [82].

## 4. Conclusions

Insights into the inhibitor–CDK6 binding mechanism and the consequential conformational alterations of CDK6 due to inhibitor associations are highly significant for the development of potent inhibitors targeting CDK6. Our present work involved conducting 2.4 μs multiple independent MD simulations of four replicates of Apo CDK6 and LQQ-, 6ZV-, and 0RS-bound CDK6 to enhance the conformational sampling. The results from the computations of RMSDs and RMSFs indicate that inhibitor associations significantly impact the structural stability and modulate the changes in the flexibility of CDK6. Furthermore, DCCMs and PCA elucidated that inhibitor binding induces alterations in the motion patterns of structural domains and affects the internal dynamics of CDK6. Binding free energy estimations, leveraging various generalized Born (GB) models and empirical parameters, underscore the pivotal role of van der Waals interactions in governing the affinity between inhibitors and CDK6. The energetic contributions from individual residues suggest that residues I19, K29, M54, P55, F98, H100, and L152 can be used as potential focal points for crafting highly efficacious CDK6 inhibitors.

## Figures and Tables

**Table 1:** Binding affinities of three small-molecule inhibitors to CDK6 computed with the MM-GBSA approach with four GB models.

Components ^a^	LQQ				6ZV				0RS			
	IGB = 1	IGB = 2	IGB = 5	IGB = 66	IGB = 1	IGB = 2	IGB = 5	IGB = 66	IGB = 1	IGB = 2	IGB = 5	IGB = 66
$∆E_{ele}$	−17.65	−17.65	−17.65	−17.65	−17.49	−17.49	−17.49	−17.49	−30.62	−30.62	−30.62	−30.62
$∆E_{vdw}$	−43.16	−43.16	−43.16	−43.16	−46.87	−46.87	−46.87	−46.87	−37.36	−37.36	−37.36	−37.36
$∆G_{gb}$	30.79	36.92	32.02	42.58	31.22	37.14	4.38	44.55	39.84	45.91	29.02	53.45
$∆G_{surf}$	−4.94	−3.43	−3.43	−3.43	−5.44	−3.78	−3.78	−3.78	−4.26	−2.96	−2.96	−2.96
^b^ $\Delta G_{\mathrm{pol}}$	13.14	19.27	14.37	24.93	13.73	19.65	−13.11	27.06	9.22	15.29	−1.60	22.83
^c^ $\Delta G_{\mathrm{hydro}}$	−48.10	−46.59	−46.59	−46.59	−52.31	−50.65	−50.65	−50.65	−41.62	−40.32	−40.32	−40.32
^d^ ΔH	−34.96	−27.32	−32.22	−21.66	−38.58	−31.00	−63.76	−23.59	−32.40	−25.03	−41.92	−17.49
−T∆S	18.53				18.20				17.37			
$∆G_{bind}$	−16.43	−8.79	−13.69	−3.13	−20.38	−12.80	−45.56	−5.39	−15.03	−7.66	−24.55	−0.12
IC_50_(nM)	16				10				300			
^e^ $\Delta G_{\exp}$	−10.7				−10.9				−8.92			

^a^ All free energy components are in scaled in kcal/mol; ^b^ $∆G_{pol}=∆E_{ele}+∆G_{gb}$, which is employed to signify the polar interactions of the inhibitors with CDK6; ^c^ $G_{hydro}=∆E_{vdw}+∆G_{surf}$, which is applied to denote the hydrophobic interactions of the inhibitors with CDK6; ^d^ $∆H=∆G_{pol}+∆G_{hydro}$, which is adopted to indicate the enthalpy effect during binding of the inhibitors to CDK6; ^e^ $∆G_{exp}$ is generated using $∆{G=-RTlnIC}_{50}$ with the experimental values of IC_50_ in references [31,56,57].

**Table 2:** Energetic contributions of the side chains and backbones of residues to inhibitor–CDK6 binding ^a^.

Inhibitors	Residues	*S_vdw_*	*B_vdw_*	*T_vdw_*	*S_ele_*	*B_ele_*	*T_ele_*	*S_gb_*	*B_gb_*	*T_gb_*	Δ*G*
	I19	−0.78	−0.18	−0.96	0.00	−0.15	−0.15	0.01	0.26	0.27	−0.93
	K29	−0.41	−0.11	−0.52	−0.59	−0.15	−0.74	0.80	0.14	0.94	−0.35
	R44	−0.01	−0.01	−0.02	−0.12	−0.06	−0.18	0.12	0.09	0.22	0.01
	M54	−0.62	−0.12	−0.74	−0.08	−0.01	−0.09	0.18	0.13	0.31	−0.59
LQQ	P55	−0.37	−0.14	−0.51	−0.03	0.06	0.03	0.10	−0.03	0.06	−0.46
	L56	−0.88	−0.13	−1.01	−0.04	−0.04	−0.09	0.10	0.17	0.26	−0.93
	F98	−0.89	−0.11	−1.01	−0.08	0.08	−0.00	0.14	0.00	0.15	−0.94
	H100	−0.61	−0.14	−0.74	−0.19	−0.51	−0.70	0.45	0.25	0.70	−0.80
	V101	−0.34	−0.22	−0.56	0.02	−0.57	−0.55	−0.01	0.62	0.61	−0.54
	L152	−0.72	−0.03	−0.75	0.02	0.05	0.08	0.00	−0.01	−0.01	−0.78
	I19	−1.59	−0.32	−1.91	−0.05	−0.17	−0.22	0.10	0.43	0.53	−1.80
	K29	−1.00	−0.23	−1.23	−1.19	−0.28	−1.47	1.58	0.31	1.88	−0.92
	R44	−0.04	−0.03	−0.07	−0.50	−0.16	−0.66	0.52	0.20	0.72	−0.01
	M54	−0.57	−0.39	−0.96	−0.05	−0.21	−0.26	0.14	0.36	0.50	−0.77
6ZV	P55	−1.16	−0.42	−1.58	−0.28	−0.27	−0.55	0.29	0.36	0.65	−1.62
	L56	−0.27	−0.16	−0.43	0.03	−0.48	−0.46	−0.00	0.25	0.25	−0.66
	F98	−0.05	−0.02	−0.07	−0.01	0.02	0.00	0.01	−0.01	−0.01	−0.07
	H100	−0.93	−0.07	−1.00	−0.27	−0.17	−0.43	0.90	0.09	1.00	−0.53
	V101	−0.32	−0.35	−0.67	0.00	0.03	0.03	0.00	0.37	0.37	−0.32
	L152	−0.80	−0.04	−0.84	−0.01	0.01	0.00	0.06	0.01	0.07	−0.86
	I19	−0.84	−0.28	−1.12	0.00	0.09	0.09	0.01	0.14	0.15	−0.98
	K29	−0.50	−0.09	−0.59	−0.95	−0.16	−1.10	1.17	0.20	1.37	−0.38
	R44	−0.26	−0.02	−0.28	−3.35	−0.36	−3.71	2.74	0.38	3.12	−0.90
	M54	−1.06	−0.37	−1.42	−0.20	−0.11	−0.31	0.29	0.21	0.50	−1.33
0RS	P55	−0.40	−0.15	−0.55	−0.00	−0.13	−0.13	0.04	0.21	0.25	−0.47
	L56	−0.39	−0.08	−0.47	−0.02	0.20	0.18	0.03	−0.17	−0.14	−0.46
	F98	−0.08	−0.03	−0.10	−0.01	0.06	0.05	−0.00	−0.05	−0.05	−0.10
	H100	−1.06	−0.10	−1.16	0.19	−0.91	−0.73	0.36	0.52	0.88	−1.08
	V101	−0.26	0.06	−0.20	−0.06	−1.25	−1.31	0.01	0.59	0.60	−0.91
	L152	−0.79	−0.07	−0.85	0.03	−0.11	−0.08	0.01	0.11	0.12	−0.89

^a^ All values are expressed in kca/mol. *S_vdw_*: the van der Waals energies (*T_vdw_*) of the side chains of the residue, *B_vdw_*: the van der Waals energies of the backbone atoms, *S_ele_*: the electrostatic energies (*T_ele_*) of the side chains of the residue, *B_ele_*: the electrostatic energies of the backbone atoms, *S_gb_*: the solvation energies (*T_gb_*) of the side chains of the residue, *B_gb_*: the solvation energies of the backbone atoms, Δ*G*: the binding energies of the residues.

**Table 3:** Hydrogen bonds formed between inhibitors and CDK6 analyzed by the program CPP TRAJ.

Complexes	Hydrogen Bonds ^a^	Distance (Å)	Angle (°)	Occupancy (%) ^b^
	R60-NE-HE···LQQ-O01	3.17	132.95	33.57
^c^ LQQ-CDK6	R60-NH2-HH21···LQQ-O01	3.10	133.81	26.31
	R60-N-H···LQQ-O01	3.15	145.22	17.97
	A30-O···6ZV-N7-H8	2.94	165.07	21.51
^c^ 6ZV-CDK6	A30-N-H···6ZV-N1	3.31	153.76	20.64
	F28-N-H···6ZV-F29	3.30	128.22	12.23
	V101-N-H···0RS-O1	2.82	149.30	27.35
	E52-OE2···0RS-N3-H8	2.84	157.67	24.81
^c^ 0RS-CDK6	D102-N-H···0RS-N4	3.26	127.43	18.40
	D102-N-H···0RS-O1	3.20	157.97	17.98
	Q103-N-H···0RS-N4	3.30	130.60	14.63

^a^ Hydrogen bonds are determined by the acceptor–donor atom distance of <3.5 Å and acceptor–H-donor angle of >120. ^b^ Occupancy (%) is defined as the percentage of simulation time that a specific hydrogen bond exists. ^c^ The full lines represent chemical bonds, and the dotted lines indicate hydrogen bonding interactions.

## Data Availability

Supporting data are supplied with the manuscript.

## Supplementary Materials

The following supporting information can be downloaded at https://www.mdpi.com/article/10.3390/molecules30050979/s1: Figure S1. Root-mean-square deviations (RMSDs) of backbone atoms in CDK6 calculated using multiple independent MD trajectories of four replicas: (A) Apo CDK6, (B) LQQ-bound CDK6, (C) 6ZV-bound CDK6, and (D) 0RS-bound CDK6; Figure S2. The functions of eigenvalues versus eigenvector indexes extracted from principal component analysis based on the single joined MSMD trajectory, which are applied to describe structural fluctuations in CDK6 along the eigenvectors; Figure S3. Free energy surfaces and representative structures of Apo CDK6: (A) free energy landscape and (B) structural superimposition of Apo CDK6 trapped at the EV1-EV4; Figure S4. Molecular structural information of LQQ-bound CDK6 located at the energy valleys (A) EV1, (B) EV2, (C) EV3, and (D) EV4; Figure S5. Molecular structural information of 6ZV-bound CDK6 located at the energy valleys (A) EV1, (B) EV2, (C) EV3, and (D) EV4; Figure S6. Molecular structural information of 0RS-bound CDK6 located at the energy valleys (A) EV1, (B) EV2, (C) EV3, and (D) EV4; and Figure S7. Molecular docking binding sites: (A) LQQ-bound CDK6, (B) 6ZV-bound CDK6, and (C) 0RS-bound CDK6.

## Abbreviations

The following abbreviations are used in this manuscript:

CDK6	cyclin-dependent kinase 6
MM-GBSA	molecular mechanics generalized Born surface area
CDKs	cyclin-dependent kinases
MESP	molecular electrostatic potential
PCA	principal component analysis
MD	molecular dynamics
RMSDs	root-mean-square deviations
SIT	single integrated trajectory
RMSFs	root-mean-square fluctuations
DCCMs	dynamic cross-correlation maps
ACMs	anticorrelated movements
FES	free energy surfaces
EV	energy valleys
