# Supplementary material for: Binding Mechanism of Inhibitors to CDK6 Deciphered by Multiple Independent Molecular Dynamics Simulations and Free Energy Predictions
Source: Molecules. 2025 Feb 20;30(5):979. doi: 10.3390/molecules30050979 (PMC11901890; doi:10.3390/molecules30050979)
Supplement: Supplementary file 1 [file molecules-30-00979-s001.zip › molecules-3407076-supplementary.pdf]

Supporting Information

Binding Mechanism of Inhibitors to CDK6 Deciphered by Multiple Independent Molecular Dynamics Simulations and Free Energy Predictions

Lifei Wang [1], Yan Wang [1], Lulu Zhang [1], Juan Zhao [1], Shiliang Wu [1] and Zhiyong Yang [2,*]

[1] School of Science, Shandong Jiaotong University, Jinan 250357, China; wanglf@sdjtu.edu.cn (L.W.); 211092@sdjtu.edu.cn (Y.W.); zhanglulu@sdjtu.edu.cn (L.Z.); zhaojuan@sdjtu.edu.cn (J.Z.); 211061@sdjtu.edu.cn (S.W.)

[2] Department of Physics, Jiangxi Agricultural University, Nanchang 330045, China

* Correspondence: zhiyongyang2009@163.com

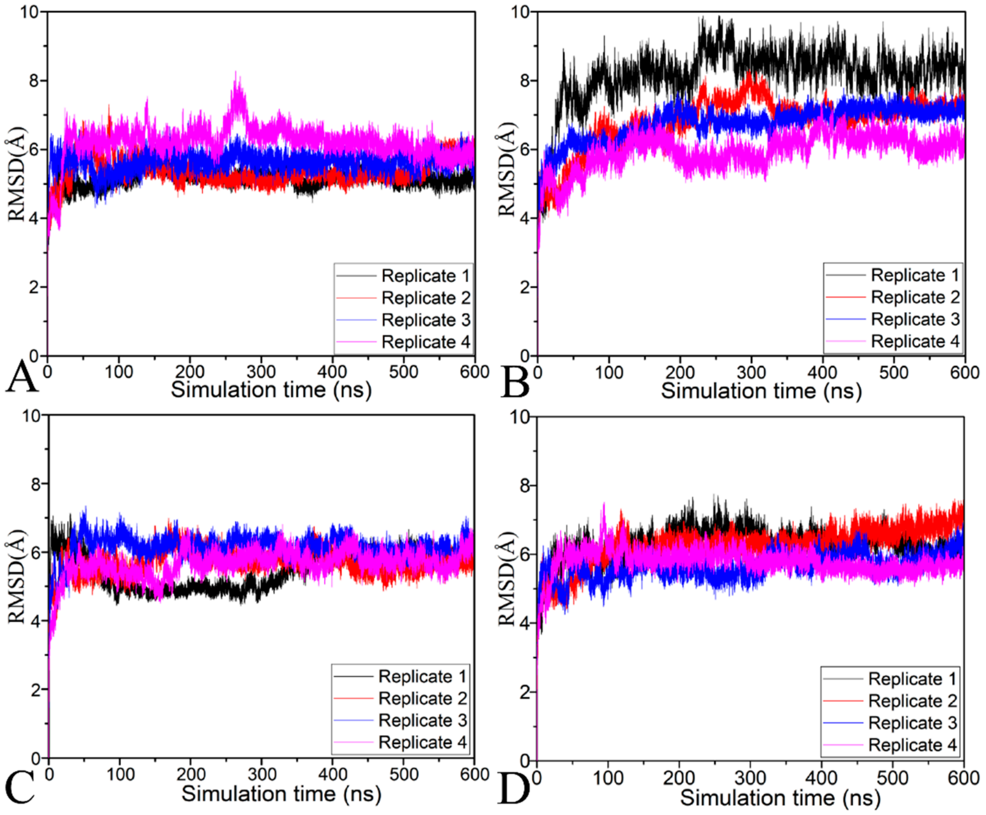

Figure. S1. Root-mean-square deviations (RMSDs) of backbone atoms in CDK6 calculated by using MIMD trajectories of four replicates: (A) Apo CDK6, (B) LQQ-bound CDK6, (C) 6ZV-bound CDK6, and (D) 0RS-bound CDK6.

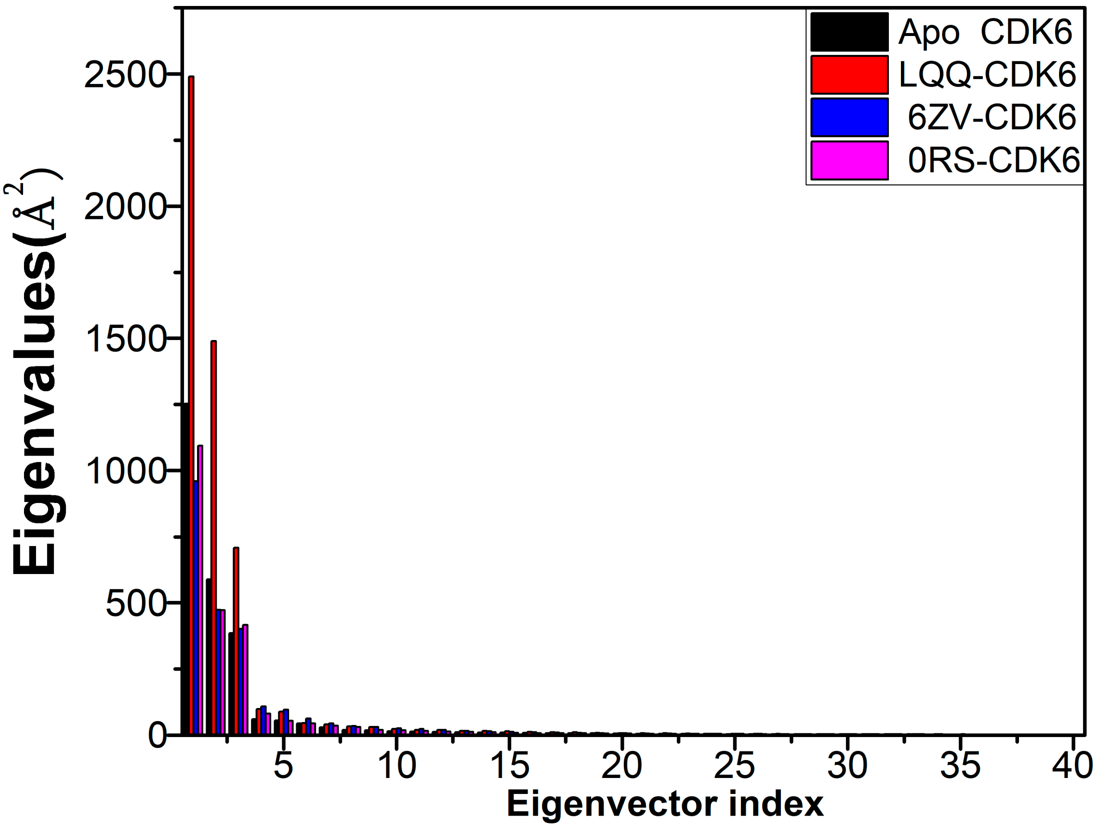

Figure S2. The function of eigenvalues versus eigenvector indexes extracting from principal component analysis based on the single joined multiple independent MD trajectory, which is applied to describe structural fluctuations of CDK6 along the eigenvectors.

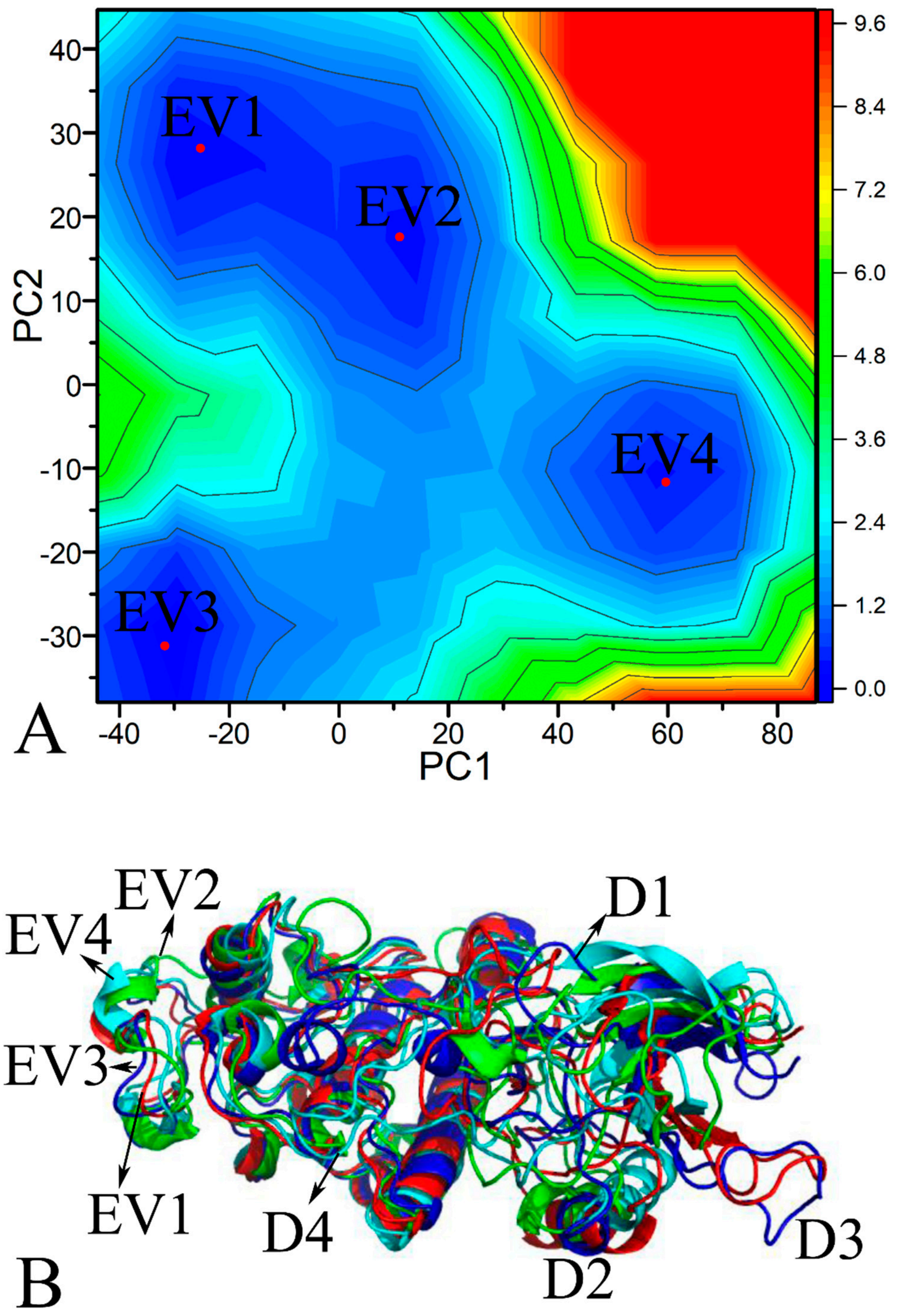

Figure S3. Free energy surfaces and the representative structures of the Apo CDK6: (A) free energy landscape and (B) structural superimposition of the Apo CDK6 trapped at the EV1-EV4.

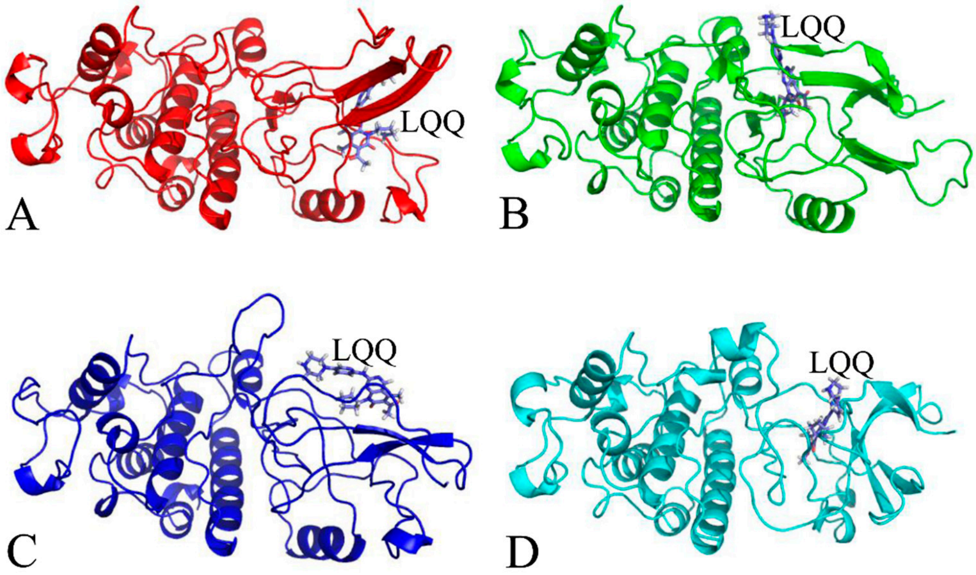

Figure S4. Molecular structural information of the LQQ-bound CDK6 located at energy valleys: (A) EV1, (B) EV2, (C) EV3, and (D) EV4.

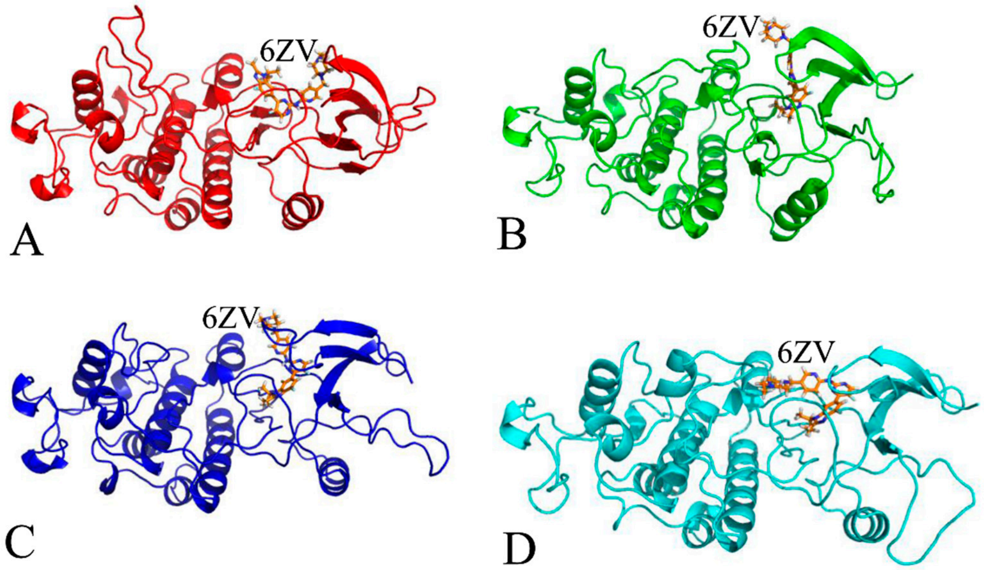

Figure S5. Molecular structural information of the 6ZV-bound CDK6 located at energy valleys: (A) EV1, (B) EV2, (C) EV3, and (D) EV4.

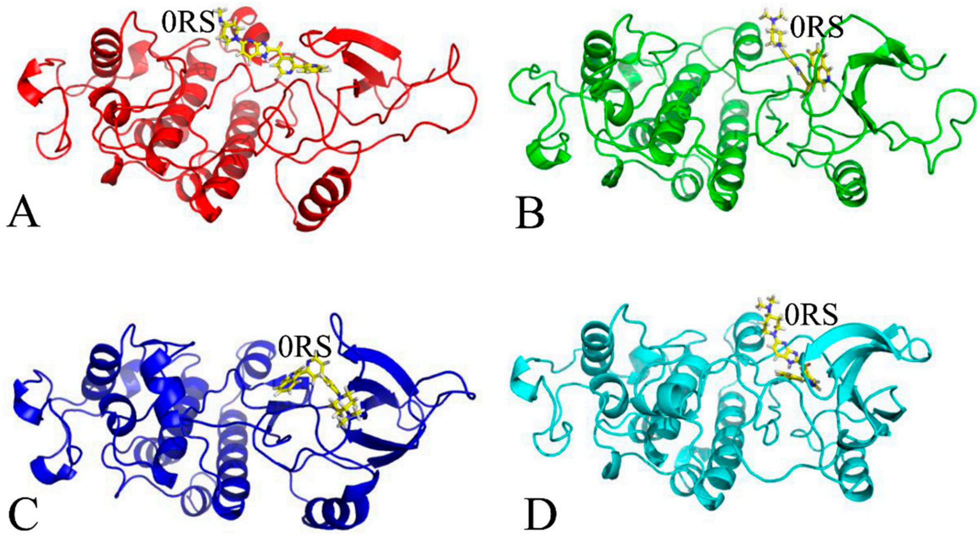

Figure S6. Molecular structural information of the 0RS-bound CDK6 located at energy valleys: (A) EV1, (B) EV2, (C) EV3, and (D) EV4.

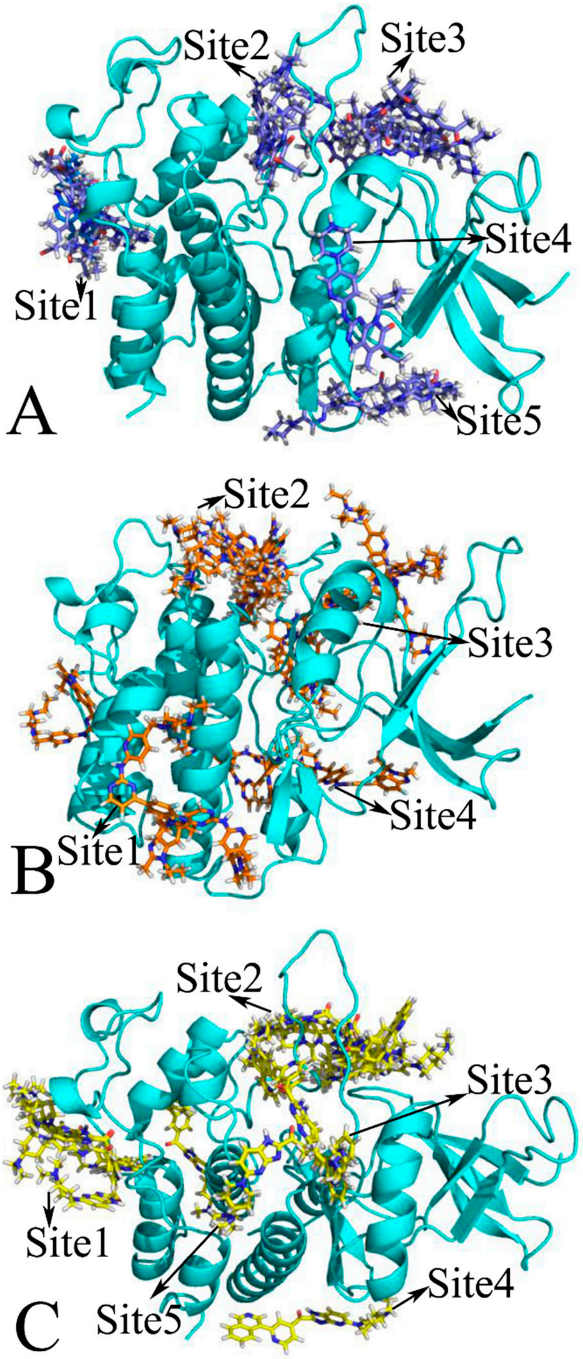

Figure S7. Molecular docking binding sites: (A) LQQ-bound CDK6, (B) 6ZV-bound CDK6, and (C) 0RS-bound CDK6.

Table S1. The parameters adopted in MM-GBSA approaches with four generalized Born models.

Parameters	IGB=1	IGB=2	IGB=5	IGB=66
γ	0.0072	0.005	0.005	0.005
β	0.00	0.00	0.00	0.00
radii	mbondi	mbondi2	mbondi2	bondi

Table S2. Binding free energies of inhibitors to CDK6 derived from molecular docking (kcal/mol).

Binding pose number	LQQ	6ZV	0RS
1	-8.0	-8.6	-7.2
2	-7.4	-8.1	-7.0
3	-7.4	-8.0	-6.7
4	-7.1	-8.0	-6.7
5	-7.0	-7.9	-6.2
6	-7.0	-7.7	-6.0
7	-6.9	-7.5	-6.0
8	-6.9	-7.4	-6.0
9	-6.9	-7.4	-6.0
10	-6.9	-7.4	-5.8
11	-6.9	-7.3	-5.8
12	-6.9	-7.2	-5.7
13	-6.7	-7.2	-5.7
14	-6.7	-7.1	-5.6
15	-6.6	-7.1	-5.6
16	-6.6	-7.1	-5.5
17	-6.5	-7.0	-5.5
18	-6.5	-6.9	-5.5
19	-6.5	-6.9	-5.5
20	-6.4	-6.9	-5.4
